# Methods for adjusting population structure and familial relatedness in association test for collective effect of multiple rare variants on quantitative traits

**DOI:** 10.1186/1753-6561-5-S9-S35

**Published:** 2011-11-29

**Authors:** Qunyuan Zhang, Doyoung Chung, Aldi Kraja, Ingrid I Borecki, Michael A Province

**Affiliations:** 1 Division of Statistical Genomics, Washington University School of Medicine, 4444 Forest Park Boulevard, St. Louis, MO 63108, USA

## Abstract

Because of the low frequency of rare genetic variants in observed data, the statistical power of detecting their associations with target traits is usually low. The collapsing test of collective effect of multiple rare variants is an important and useful strategy to increase the power; in addition, family data may be enriched with causal rare variants and therefore provide extra power. However, when family data are used, both population structure and familial relatedness need to be adjusted for the possible inflation of false positives. Using a unified mixed linear model and family data, we compared six methods to detect the association between multiple rare variants and quantitative traits. Through the analysis of 200 replications of the quantitative trait Q2 from the Genetic Analysis Workshop 17 data set simulated for 697 subjects from 8 extended families, and based on quantile-quantile plots under the null and receiver operating characteristic curves, we compared the false-positive rate and power of these methods. We observed that adjusting for pedigree-based kinship gives the best control for false-positive rate, whereas adjusting for marker-based identity by state slightly outperforms in terms of power. An adjustment based on a principal components analysis slightly improves the false-positive rate and power. Taking into account type-1 error, power, and computational efficiency, we find that adjusting for pedigree-based kinship seems to be a good choice for the collective test of association between multiple rare variants and quantitative traits using family data.

## Background

Because of the limitation of single-nucleotide polymorphism (SNP) array-based genotyping technology in detecting rare genetic variants, genome-wide association scans usually focus only on common variants analysis. Advances in DNA sequencing technologies in recent years, however, have allowed the identification and genotyping of rare variants with substantially higher accuracy and rapidly decreasing cost, which makes it practicable to detect rare variants in relatively large populations and thus enables association scans of these variants for human complex diseases. Facilitated by these technologies, investigators have recognized rare variants as one important factor that contributes to human complex-disease-related traits and interest in them is increasing [1-8].

Statistically, because of the extremely low frequency of rare variants in populations, the power of detecting disease-associated individual rare variants usually is poor. To increase the power, investigators have proposed a useful strategy in which multiple rare variants are collapsed into one variant; then, the collective effect of multiple rare variants is tested rather than individual rare variants. Different collapsing methods have been developed [9-14]. Besides the statistical methods, family data provide another additional resource that may add more power in the association analysis of rare variants, because functional rare variants for a specific trait could be more enriched in some families than in populations. However, when family data are used for association analysis, both population structure and familial relatedness between individuals need to be addressed and adjusted to obtain unbiased statistical results. Although linkage-based and transmission-based methods are commonly used for the analysis of family data with no need for adjusting for population structure and familial relatedness, they are not applicable to collapsing tests of the collective effect of multiple rare variants. Therefore most of the previous studies of collective tests of rare variants were focused on binary traits from case and control data, and methods for detecting rare variants collectively associated with quantitative traits using family data have not been well established.

To exploit advantages from both collective tests and family data, we propose to combine a collapsing strategy with the framework of the unified mixed model (UMM) [15] for the association analysis of rare variants and quantitative traits. The UMM methods were developed primarily for common variant analysis in genome-wide association scans; most of them, however, have not been investigated and validated in the context of rare variants. Here, we borrow the idea from the UMM and present a comparison of several possible methods under the framework of the UMM, with an emphasis on statistical properties of collective association tests of multiple rare variants and quantitative traits using family data.

## Methods

### Models

To adjust for both population structure and familial relatedness in association analyses, we use the following linear mixed model:

*Y* = *Xb* + *Qv* + *Zu* + *e*, (1)

where *Y* is the quantitative trait of interest, *X* is the genotype data, *b* is the fixed effect(s) of genotype(s) under the test, *Q* is the population structure variables, *v* is the fixed effects of *Q*, u is the random polygenic effects of individuals, Z is the design matrix of u, and *e* is the random residual error.

Equation (1) was originally proposed as the UMM for association analysis of common variants [15], in which *Q* and *Z* were incorporated to adjust for population structure and familial relatedness, respectively, and *X* was usually the genotype data for individual SNPs. Here, we propose to combine the UMM with a collapsing test of rare variants by replacing *X* with the collapsing variable of multiple rare variants from a given genetic unit. There are different ways to collapse multiple rare variants; here, we choose Li and Leal’s method [10].

Our goal is to compare six different methods under the framework of the UMM for the collapsing test: (1) a simple regression with no adjustment for population structure and familial relatedness, denoted REG; (2) adjustment for population structure using principal components of genotypes, denoted PC; (3) adjustment for familial relatedness using a pedigree-based kinship matrix, denoted KIN; (4) adjustment for population structure and familial relatedness using the PC and KIN methods, denoted PC-KIN; (5) adjustment for familial relatedness using a marker-based identical-by-state kinship matrix, denoted IBS; (6) adjustment for population structure and familial relatedness using the PC and IBS methods, denoted PC-IBS. More details about the six methods are presented in Table 1.

**Table 1:** Six methods for comparison

Method	Model	Detail
REG	*Y* = *Xb* + *e*	Ignoring population structure and familial relatedness, with no adjustment
PC	*Y* = *Xb* + *Qv* + *e*	Top 10 eigenvectors from principal components analysis are used as the input of *Q*
KIN	*Y* = *Xb* + *Zu* + *e*	Kinship matrix based on pedigree data is used as *Z* to define covariance structure of *u*
PC-KIN	*Y* = *Xb* + *Qv* + *Zu* + *e*	Combining PC and KIN methods
IBS	*Y* = *Xb* + *Zu* + *e*	IBS matrix based on genotype data is used as *Z* to define covariance structure of *u*
PC-IBS	*Y* = *Xb* + *Qv* + *Zu* + *e*	Combining PC and IBS methods

### Data and computation

We used the 200 replications of data of 697 subjects from 8 extended families simulated from Genetic Analysis Workshop 17 (GAW17) [16]. We chose the quantitative trait Q2 to compare the methods and as the target trait (i.e., *Y* in the models). For each gene, we collapsed the genotypes with minor allele frequency (MAF) less than 0.01 into a binary (1 or 0) variable according to the presence or absence of at least one rare variant in a subject, based on Li and Leal’s method [10]. We then used this binary variable as the collective predictor variable of interest (i.e., *X*) in the models.

We obtained the top 10 eigenvectors for the 697 subjects using Eigenstrat 2.0 [17] using all SNPs with MAF > 10% and then fitted these eigenvectors into the PC-related models (PC, PC-KIN, and PC-IBS) as fixed-effect population structure covariates (i.e., *Q*). Using the R package Kinship 1.10-23 (http:// cran.r-project.org/web/packages/kinship), we obtained the kinship matrix from the pedigree information and fitted it as *Z* into the models. Using 5,000 randomly selected makers with MAF > 0.05 and the R package EMMA 1.1.2 [18], we obtained the IBS matrix and then fitted it as *Z* into the models.

To estimate parameters and perform significance tests, we used SAS 9.2 for the REG and PC models, Kinship 1.10-23 for the KIN and PC-KIN models, and EMMA 1.1.2 for the IBS and PC-IBS models. Quantile-quantile (Q-Q) plots under the null and receiver operating characteristic (ROC) curves were investigated and compared between the six models.

## Results

We compared the six selected methods in terms of two important statistical properties, false-positive rate (FPR) and power, by visualization of Q-Q plots and ROC curves.

**Figure 1:**
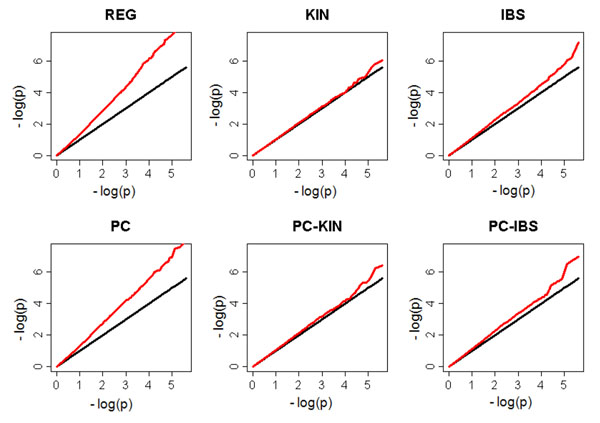
**Q-Q plots for six different methods.** Q-Q plots of −log_10_ scaled *p*-values for six different methods based on 1,940 genes from 697 subjects (8 extended families) and 200 replications of quantitative trait Q2 simulated by GAW17 under the null hypothesis. Red curves, observed; black curves, expected.

Q-Q plots (Figure 1) show that the REG model ignoring population and family structure in the data results in a significant FPR. Both the KIN and IBS models reduce FPR significantly, whereas the KIN model slightly outperforms the IBS model. The effects of adjusting for population structure using principal components in all models are insignificant.

**Figure 2:**
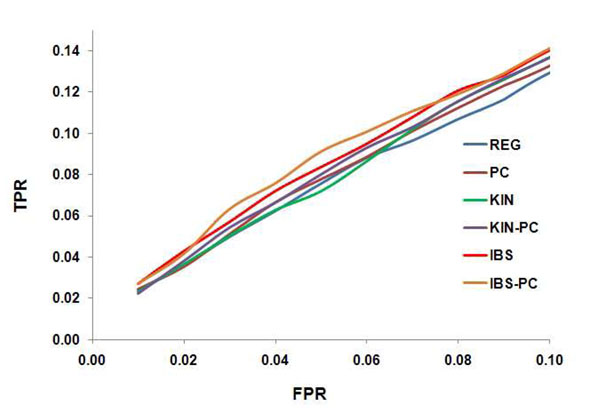
**ROC curves for six different methods.** ROC curves for six different methods based on 13 genes from 697 subjects (8 extended families) and 200 replications of quantitative trait Q2 simulated by GAW17. FPR is limited to be less than 0.1 because in practice only the true-positive rate (TPR) with a low FPR is of interest.

According to the ROC curves (Figure 2) and in terms of detecting the collective effect of multiple rare variants in a gene, the IBS model has the best power and the REG model the lowest; the KIN model is close to the IBS model. Adjustment for principal components for most analyses slightly increases the power.

## Discussion and conclusions

By combining FPR and power, we find that the KIN and IBS models outperform other methods for the rare variant collective association test. The two methods have pros and cons. The KIN model is computationally more efficient than the IBS model but needs pedigree information. The IBS model requires no extra pedigree information but may be computationally intense when the sample size is large. Therefore the KIN model should be a good choice when pedigree information is available, especially for studies with large sample size and strong family structure.

The result of PC adjustment for rare variant analysis is inconsistent with that for common variant analysis [17]. An important reason could be that the top 10 eigenvectors we used capture only about 15% of the variation of genotypes. Another possible reason is that the GAW17 variant data are from only one chromosome (and some selected genes), and the principal components based on these variants may not be able to represent the genetic background well.

The collapsing method we used here assumes that all the casual variants from a gene have the same effect direction, which may not be true. Some effect direction-sensitive methods can be used, but they usually require a permutation test. Because permutation will destroy family structure, it is still an open question of how to perform the permutation test on family data.

## Competing interests

The authors declare that there are no competing interests.

## Authors’ contributions

QZ carried out the statistical analysis and drafted the manuscript. DC participated in the programming of statistical methods. AK prepared the data and assisted in computation. IIB and MAP participated in the design of the study and helped to organize the structure of the manuscript. All authors read and approved the final manuscript.
